# An Energy Aware Adaptive Sampling Algorithm for Energy Harvesting WSN with Energy Hungry Sensors

**DOI:** 10.3390/s16040448

**Published:** 2016-03-28

**Authors:** Bruno Srbinovski, Michele Magno, Fiona Edwards-Murphy, Vikram Pakrashi, Emanuel Popovici

**Affiliations:** 1 Department of Electrical and Electronic Engineering, University College Cork, College Road, Cork T12 YN60, Ireland; f.edwardsmurphy@umail.ucc.ie (F.E.-M.); e.popovici@ucc.ie (E.P.); 2 MaRine Renewable Energy Ireland (MaREI), Environmental Research Institute, University College Cork, College Road, Cork T12 YN60, Ireland; V.Pakrashi@ucc.ie; 3 Department of Information Technology and Electrical Engineering, ETH Zurich, Zürich 8092, Switzerland; magnom@iis.ee.ethz.ch; 4 Department of Electrical, Electronic and Information Engineering (DEI), University of Bologna, Bologna 40126, Italy; 5 Dynamical Systems and Risk Laboratory, School of Engineering, University College Cork, College Road, Cork T12 YN60, Ireland

**Keywords:** adaptive sampling, energy harvesting, energy management, power hungry sensors, solar energy harvesting, wind energy harvesting, WSN

## Abstract

Wireless sensor nodes have a limited power budget, though they are often expected to be functional in the field once deployed for extended periods of time. Therefore, minimization of energy consumption and energy harvesting technology in Wireless Sensor Networks (WSN) are key tools for maximizing network lifetime, and achieving self-sustainability. This paper proposes an energy aware Adaptive Sampling Algorithm (ASA) for WSN with power hungry sensors and harvesting capabilities, an energy management technique that can be implemented on any WSN platform with enough processing power to execute the proposed algorithm. An existing state-of-the-art ASA developed for wireless sensor networks with power hungry sensors is optimized and enhanced to adapt the sampling frequency according to the available energy of the node. The proposed algorithm is evaluated using two in-field testbeds that are supplied by two different energy harvesting sources (solar and wind). Simulation and comparison between the state-of-the-art ASA and the proposed energy aware ASA (EASA) in terms of energy durability are carried out using in-field measured harvested energy (using both wind and solar sources) and power hungry sensors (ultrasonic wind sensor and gas sensors). The simulation results demonstrate that using ASA in combination with an energy aware function on the nodes can drastically increase the lifetime of a WSN node and enable self-sustainability. In fact, the proposed EASA in conjunction with energy harvesting capability can lead towards perpetual WSN operation and significantly outperform the state-of-the-art ASA.

## 1. Introduction

One of the major obstacles for Wireless Sensor Networks (WSN) technology to expand even further and ultimately replace, wherever possible, wired sensor solutions, is their limited energy availability, which significantly reduces their lifetime, as batteries are the primary energy source for WSN nodes. The rate of improvement in battery capacity in the last decades has not increased significantly [1], when compared to advances in processing, sensing, and memory technologies. However, novel applications as well as miniaturization requirements put significant constraints on energy management systems. To that end, industrial and academic researchers are trying to explore several innovative solutions while waiting for a breakthrough in energy storage technology. The most successful approaches are to develop more energy efficient designs (for both hardware and software), [2,3] and also to harvest energy from the environment often exploiting heterogeneous sources [4] in order to satisfy the energy needs for prolonged periods of time.

Harvested energy is variable in time and not easily predictable. Low levels of available energy might also affect the fidelity and accuracy of the sensed/processed data, and might significantly impact on the reliability of the communication [5]. During the last two decades, research in WSN has been extensively focused on developing different Medium Access Control (MAC) and routing protocols, with the ultimate aim of effectively managing the activity of the transceiver, based on the assumption that data acquisition and processing have significantly lower energy consumption than wireless communication [6]. However, there are many applications where it can be wrong to assume that this is true. For instance, in cases of structural health monitoring (SHM) of civil infrastructures, the latest generation of sensors used by geomonitoring experts are extremely expensive with respect to energy requirements. In some applications, the energy cost of sensor sampling ranges from approximately double, up to few orders of magnitude more, than the energy required to wirelessly transmit data [7]. This is due to the fact that some of the sensors (e.g., gas and pressure sensors) have an associated warm up period, or that measurement of the phenomenon requires sampling in the range of seconds. In such applications, where the acquisition times are longer than data transmission, the energy consumption of the sensor is often higher than the energy spent for data transmission, which demands the MAC protocols to be complemented with techniques that implement an efficient energy management mechanism for the sensors [6].

In this paper, we present a novel energy aware adaptive sampling (EASA) approach that combines an adaptive sampling algorithm (ASA) with an energy management technique optimized for energy harvesting WSN. The main goal of this work is to show that combining energy harvesting from the environment and effectively managing node activity (*i.e.*, the sampling rate of the sensors) according to the energy levels and the dynamics of the phenomenon observed, can ensure an enhanced lifespan of the nodes in the network, thereby achieving self-sustainability. The suggested energy aware adaptive sampling method allows each node in the network to adapt the sampling rate to its sensors according to the energy available in the system. The model proposed for adjusting the sampling rate, and the proposed power model of an EH WSN node, are designed to be generic and easy to implement with any specific WSN platform. Data collected from two different deployments are used to evaluate the proposed approach. Two different scenarios with different power hungry sensors and with different sources for harvested energy are used to collect real field data. In the first deployment (I), wind speed and direction are measured with power hungry ultrasonic wind sensors that are connected to a custom made platform based on a Texas Instruments MSP430 microcontroller and a CC2520 radio transceiver. The platform is equipped with a micro wind generator for harvesting energy from the wind [8]. In the second deployment (II), data are collected with an off the shelf platform, interfaced with power hungry gas sensors used to monitor the conditions within a beehive [9]. This platform uses a solar panel to harvest energy from the sun and five different gas sensors that require long warm up periods. Detailed analysis and measurement of the energy consumption of the embedded systems are performed with the same firmware implemented on the deployments. This allows identification of the energy consumed by the system during various active states and replication of the in-field system behavior. In this way, a precise energy model is developed that is later used in the simulations.

The proposed algorithm is implemented in MATLAB using the real sensor power consumption and the harvested energy collected from the two deployments. This allows comparison with the traditional adaptive sampling algorithm to be done [6]. The contributions of this paper are as follows:

- An energy aware ASA optimized for energy harvesting nodes achieving self-sustainability with power hungry sensors is presented.
- Precise energy model for a WSN network with power-hungry sensors and energy harvesting capabilities.
- Evaluation of the proposed approach within two application domains, namely SHM and monitoring of beehives.
- Simulation evaluation using long term in-field measurements and comparison with the ASA (without energy awareness) and the fixed sampling rate (conventional method) in terms of energy durability.

## 2. Related Work

Energy management techniques to achieve long term monitoring and self-sustainability in WSN applications are widely used and explored in the literature at both node and network level [10,11]. The most common approaches employ: communication power management [12], voltage scaling [5], adaptive IC power modes, duty cycling [13], wake up receivers [14] and data aggregation [15]. Beside them, another popular way to reduce energy consumption in WSN is using the adaptive radio frequency approach [16,17] and also static or dynamic packet length optimization, in order to save energy on transmission [18,19]. Another approach is the implementation of adaptive sampling algorithms [6,20,21], which allows a high increase in lifetime. Today, the traditional methods, with fixed sampling rate, are replaced with solutions that modify the sampling rate of the sensors according to the needs (*i.e.*, temporal changes and frequency) of the phenomenon observed. In this way, the sensor node does not waste energy sampling the sensor more than the actual maximum frequency of the signal [20]. However, using only adaptive sampling can guarantee an extension of the lifetime, but may not guarantee self-sustainability (or energy neutrality) of WSN, as the battery is still a finite energy resource.

Energy harvesting (EH) for WSN has been very prolific in the last decade of research [22,23], trying to overcome the battery limitation. EH plays an important role in enhancing the life span of a node in a network that aims to achieve self-sustainability [2,21]. A good example of an energy harvesting system that utilizes small wind turbine to harvest wind energy and to power the associated electronic circuitry as well as sensing the wind is presented in [24]. The authors give a detailed description, analysis, and evaluation of the proposed energy harvesting system for autonomous wind sensors and prove the concept of fully autonomous self-powered wireless sensing. A similar example of an autonomous self powered WSN node is analyzed and developed in [25], where the authors consider the application of monitoring wildfire spread in a remote area. As emphasized in [25], the main problem of a battery powered WSN node in fire hazard monitoring applications is that when the battery is running low the sensor nodes could stop functioning and expose the area to fire danger, which is not acceptable for such a monitoring system. To that end, authors are suggesting a solution in the form of a self powered WSN node that will measure the wind speed at the site, and at the same time, harvest the energy from the wind to sustain the remote sensing operation of the WSN node. A battery-less self powered wireless sensor, that autonomously performs a measurement of air temperature and velocity and transmits data to an external receiving unit is presented in [26]. The proposed sensor is powered by a harvesting system that exploits the airflow. The sensor continuously operates for airflow speeds greater than 3 m/s. For slower flows, the sensor is off, and the receiver assumes that the velocity of air is below the threshold. This type of application allows the battery constraints to be overcome by excluding the battery from the system.

However, the main issue with the approach presented in [24,25,26], is the unpredictable variable nature of the energy sources in both space and time. Hence, energy harvesting systems on their own cannot ensure that the node will last [27]. Complementing the energy harvesting with an efficient energy management algorithm is often a solution to this problem [27]. An effective power management algorithm for sensor networks with sensors that do not require a high sampling rate is presented in [28]. Another interesting approach is described in [29], where the authors present an algorithm that adaptively adjusts the processor speed to achieve system-wide energy efficiency based on the workload and available energy information. In [30], the authors present an energy management technique for WSN with solar energy harvesting. They propose two adaptive transmission schemes, in which they adjust the transmission delay based only on the battery percentage and output voltage of the solar panel. However, the authors do not present any data or model of the harvested energy and the consumed energy by the platform used. In addition, the proposed approach is only evaluated on one platform.

In many WSN examples in the literature, adaptive sampling algorithms are used only as a tool to minimize the communication between the sensor node and the base station [20]. However, dynamically adapting the sampling frequency of a power hungry sensor according to the maximum frequency of the signal (which depends on the phenomenon observed), one can effectively reduce the power consumption of the sensor and at the same time reduce the radio activity. In this paper, we extend this vision by enhancing the algorithm with a power aware functionality, and verify the concept in simulation with two different deployment scenarios and different types of energy harvester. To the best knowledge of the authors, this is the first paper that presents this approach supported by simulation with in-field long term experimental evaluation. The algorithm proposed Energy Aware ASA (EASA) can be useful especially for wireless sensor nodes equipped with energy harvesting capabilities, where the battery is periodically recharged from an environmental sources.

## 3. Energy Aware Adaptive Sampling Algorithm

Typically ASA algorithms evaluate the maximum frequency (*f_Max_*) of the signal and decide the sampling frequency (*f_Sampling_*), which is usually at least twice *f_Max_* to avoid aliasing. An example of ASA algorithm (Algorithm 1) is presented in the pseudo code below and more details can be found in [6,20]:
**Algorithm 1** An example of ASA algorithm.Store an initial number of sensor samples (*W*);Estimate the *f_Max_* on the acquired *W* samples;Based on *f_Max_* set the sampling frequency to a value *f_Sampling_* = *c f_Max_* where *c* is a confidence parameter that has to be ≥ 2 to avoid aliasing;Define upper and lower frequency borders to detect the change in the maximum frequency *f_Up_* and *f_Down_* as: *f_Up_* = *min* {(1 + *δ*)*f_Max_, f_Sampling_*/2}; *f_Down_* = (1 − *δ*) *f_Max_*;*h*_1_ = 0; *h*_2_ = 0; *i* = *W* + 1;*while*(1){Acquire a sample and add to the dataset;Estimation of the current maximum frequency *f_Curr_* on the sequence (*i − W* + 1, *i*);Count the consecutive number of events when the frequency was higher or lower than previously*If* (|*f_Curr_ − f_Up_*|< |*f_Curr_ − f_Max_*|)*h*_1_*++*; *h*_2_ = 0;*else if* (|*f_Curr_ − f_Down_*|< |*f_Curr_ − f_Max_*|)*h*_2_++; *h*_1_ = 0;*if* ((*h*_1_ > *h*) || (*h*_2_ > *h*)){*f_Sampling_* = *c f_Curr_; f_Max_* = *f_Curr_*;*f_Up_* = *min*{(*1* + *δ*)*f_Max_, f_Sampling_*/2}; *f_Down_* = (1 − *δ*) *f_Max_*}}

The relevant parameters are as follows:

- *h* is the consecutive number of samples after which the algorithm detects a change. A low value of *h* indicates variation in the maximum frequency of the detected signal. As a consequence, false detections are more frequent, which finally results in a very frequent update of the sampling rate—*f_Sampling_*.
- *δ* is the minimum percentage change in the maximum frequency that must be detected by ASA.

Using the ASA algorithm can drastically reduce the number of acquired samples with respect to the traditional fixed sampling rate approach, hence saving energy [6,20]. However, to guarantee self-sustainability when the node is equipped with energy harvesters, the ASA needs to monitor the energy level of the node and manage the node activity based on the available energy. Therefore, to further increase the life span of a node and ultimately achieve self-sustainability, we propose an approach in which each of the nodes in the network will have the ability to further adapt the sampling rate of the sensors according to the energy available in the system. This approach can be used as standalone extension for every ASA algorithm. The sampling rate of the sensors refers to how often the sensors are sampled and how often the measured data are sent to the base station (BS). It is also assumed that each of the nodes in the network is able to monitor its own energy level, and is able to harvest energy from the environment. Based on their energy levels, the nodes can adjust the sampling rate received from the BS following the equations below:

(1) $$f_{EASA}=f_{Sampling}K$$

(2) $$\{\begin{array}{c} K=1\;,\;if\;E_{batt}\geq X\\ K=1-{\left(\frac{X_{level}\;-\;E_{batt}}{100}\right)}^{m\;},if\;E_{batt}<X \end{array}$$

where *K* is the function cost for the energy awareness; *E_batt_* is the battery level expressed in percentage (0–100); and *X_level_* is the selected critical level of the battery in percentage, where the sample frequency starts to decrease from *f_Sampling_* to *f_EASA_* and the system starts to conserve energy. The rate of saving is also dictated by the parameter *m*, which in our simulations was fixed to 1, 0.5, and 0.33. The proposed algorithm follows *f_Sampling_* until the energy in the battery drops below a level *X_level_*. Afterwards, *f_Sampling_* is decreased following the trend defined by Equation (2).

Using this approach can cause aliasing to occur in which case the *Nyquist* criterion (*f_sampling_* > 2 *Bandwidth*) will not be met. In this case, the sensor signal will be sub-sampled, with a frequency lower than the maximum frequency of the signal, introducing errors in the measurement. Some of these errors can be filtered as part of back end post-processing, or accepted as a trade off for staying active in the network. Further discussion on this problem is presented in Section 6.

### Energy Model of an Energy Harvesting WSN Node with a Power Hungry Sensor

The development of an appropriate energy model, capable of accurately evaluating the energy consumption of hardware in different states of operation is an important task that will make the simulation and evaluation of potential deployments possible.

The energy consumed during an operation is a function of the time required to carry out the operation in the particular operational mode of the underlying hardware. For example, once it is known that a particular task requires a certain number of microcontroller clock cycles, the amount of energy required (*E_ALG_*) is easily calculated (e.g., *E_ALG_ = P_MCU._t_ALG_*) where *P_MCU_* is the power consumption of the MCU in Active Mode, and *t_ALG_* is the time required to execute the algorithm.

**Figure 1:**
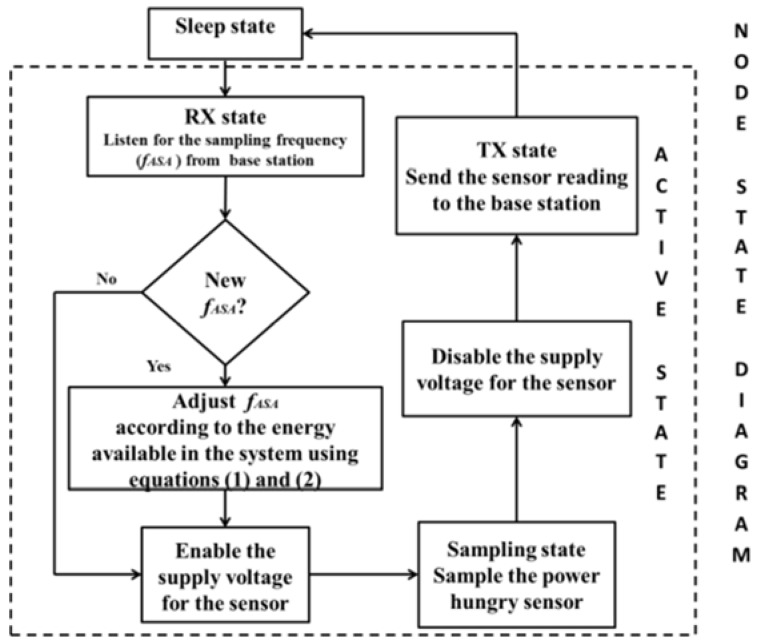
State flow diagram of a duty cycled node that has harvesting capabilities and uses a power hungry sensor-deployment (I).

In the state machine in Figure 1, we assume a duty cycled node which has harvesting capabilities and a power hungry sensor. When the node samples the sensor it also sends the measurement to the BS. We assume that the earlier described ASA is running on the BS. When the ASA detects a change in the frequency of the signal, the base station sends a new sampling frequency, *f_ASA_*, to the node. The node, which has information on how much energy is available in its battery (and how much energy is harvested), adapts this sampling frequency *f_ASA_* (received from the BS) according to Equations (1) and (2) resulting in a new sampling rate *f_EASA_*.

Assuming that the information on the harvested power available, *P_i HARVESTED_*, for each *i* energy source, and the period of harvesting is available (*t_iharv_*), the amount of harvested energy from the *n* sources can be calculated using Equation (3).

(3) $$E_{H}=\;\overset{n}{\underset{i=1}{\sum }}P_{i\;HARVESTED\;i}t_{i\;harv}$$

During the period when the node is active (*t_ACTIVE_*), the node first listens for a message that contains the new sampling frequency from the BS (*t_RX_*). The associated power consumption to this state is *P_RX_*. If within this time (*t_RX_*), the node receives a message containing a new sampling frequency, it executes the energy aware function described with Equations (1) and (2) and adapts the sampling frequency received from the BS according to its energy levels. In the case when the node does not receive a message from the BS, the node continues sampling at the previous sampling frequency. The node samples the sensor for a period of time *t_SnesorSamp_*. In the case of a power hungry sensor, the energy consumption in that period can be dominant due to the power required for sampling *P_SensorSampling_*, due to the duration of this phase, or in some cases both. After the data is acquired from the sensor, the next step is the processing phase where the data is analyzed by the microcontroller or additional circuits (*i.e.*, ADC, level translators, *etc.*). This phase is represented by *P_PROC_* and *t_PROC_* in Equations (4) and (5). Finally, the node sends a message to the BS (*t_TX_* and *P_TX_*) containing the acquired measurement. Thus, the active period can be described using Equations (4) and (5).

(4) $$t_{ACTIVE}=t_{TX}\;+\;t_{RX}+t_{SensorSamp}+t_{PROC}$$

(5) $$E_{ACTIVE}=P_{TX}t_{TX}+P_{RX}t_{RX}+P_{SensorSamp}t_{SensorSamp}+P_{PROC}t_{PROC}$$

To evaluate the system energy spent during the sleep period, Equation (6) can be used, where *P_SLEEP_* is the power consumption in sleep mode and the time spend in sleep (*t_SLEEP_*) is the total period *T* minus the activity period (*t_ACTIVE_*). Finally the total energy available in the system at each time (*t*) is defined by Equation (7) where *E_LEVEL_* is the energy available in the battery:

(6) $$E_{SLEEP}=P_{SLEEP}t_{SLEEP}$$

(7) $$E_{LEVEL}\left(t+1\right)=E_{LEVEL}\left(t\right)-E_{ACTIVE}\left(t\right)-E_{SLEEP}\left(t\right)+E_{H}\left(t\right)$$

It is important to emphasize that for the proposed energy model of an energy harvesting WSN with energy hungry sensors to be used for theoretically modeling of any harvesting system, it is enough to know the amount of harvested power, the period of harvesting and the overall consumed energy of the system during the same period.

## 4. Experimental Setup and Node Architecture

In order to evaluate the proposed energy aware algorithm, two different platforms have been used and deployed to collect field data. In the first deployment (I), a custom made platform was developed and used for monitoring the wind speed and direction with harvesting energy from the wind. In the second deployment (II) an off the shelf WSN platform was used for monitoring the gas levels (with an emphasis on measuring the CO_2_ levels) in a beehive with solar energy harvesting. The deployments are carried out separately in two different application scenarios, with different durations in order to assess the performance of the proposed algorithm in different conditions (*i.e.*, different platform, application space, and sensors).

**Figure 2:**
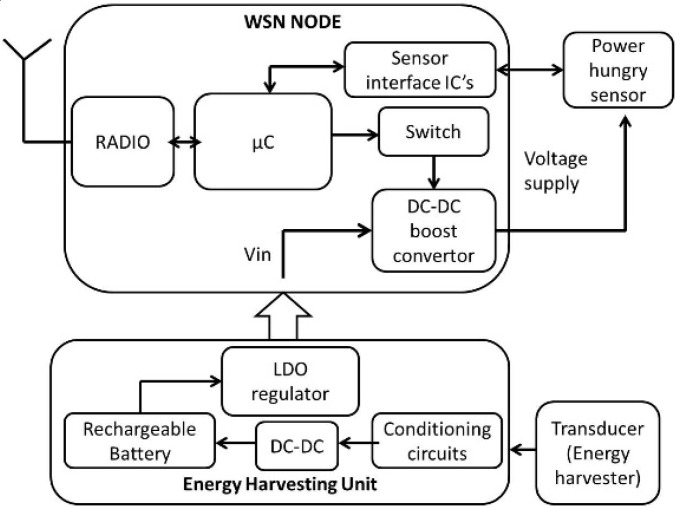
Generic block diagram of a WSN platform with an energy harvesting power unit and power hungry sensor.

A generic block diagram of the systems deployed is presented in Figure 2. In the following subsections, the deployments are described in detail.

### 4.1. Deployment (I)

**Figure 3:**
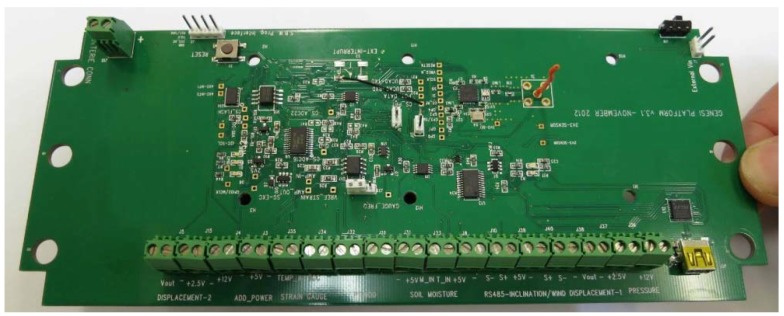
Custom made platform for deployment (I).

The custom made platform used in deployment (I) (Figure 3) is capable of supporting a variety of digital and analog sensors intended for structural health monitoring including the previously mentioned ultrasonic wind speed and direction (*WMT52*) sensor. The node uses the Texas Instruments MSP430F5437A microcontroller and the CC2520 as radio transceiver. The selected microcontroller has a 16 bit RISC CPU and offers various sleep modes with low power consumption. It also has sufficient SRAM and flash memory to accommodate the requirements of the application. The CC2520 exhibits fast wake-up times, low power consumption (two different sleep modes), and compliance with the updated IEEE802.15.4-2006 Standard.

The selected wind sensor is an ultrasonic Vaisala WMT52 sensor that has an array of three equally spaced ultrasonic transducers on a horizontal plane. The wind speed and the wind direction are determined by measuring the time it takes the ultrasound to travel from each transducer to the other two. The sensor can be interfaced using the RS-485 protocol, which requires an additional differential transceiver (level translator) in order to be interfaced to the microcontroller. A step up DC-DC converter from Analog Devices ADP2371 (3.3 V to 12 V) was used to provide the supply voltage to the wind sensor. To completely turn off the supply voltage for the sensor when not in use, a simple transistor based switch controlled by the MCU was implemented. This ultimately reduced the sleep current of the platform to 15 μA. A harvester power unit was used to harvest the wind energy with a scaled wind turbine. The harvester is a high efficiency power unit that can recharge a battery with harvested energy from the wind [31]. An off the shelf small size scaled wind turbine was used with radius of the blades of 0.17 m. This wind turbine provides a rectified (DC) output voltage that is regulated (stepped up or down) by the harvesting unit and fed to a rechargeable battery. To directly supply the node, the voltage from the battery is then further regulated to 3.3 V.

The experiment for collecting the wind speed data was carried out over a period of 35 days. The node and the wind sensor were deployed on a roof-top of a 20 m tall building located in Cork, Ireland. The node was duty cycled, sending the collected wind data every 20 s (0.05 Hz) to a base station that was connected to a laptop storing the messages in a file. The wind sensor was configured to sample with a fixed sampling frequency of 1 Hz over a period of 10 s and sending the minimum, maximum and the average value of the speed and direction within those 10 s. The 35-day data set of continuous wind sensor monitoring was used to evaluate the proposed approach in simulation.

### 4.2. Deployment (II)

The second deployment was carried out as a part of a research activity in smart monitoring of beehives [9]. The main goal of this research is to obtain knowledge of the conditions within the hive, and use this knowledge to inform the beekeeper, with the aim of maintaining a healthy hive through the use of heterogeneous WSN and mobile technologies [9]. The same deployment is also used for weather prediction [9].

A deployment period of 14 days has been used in this paper to evaluate the benefits of the proposed approach. The critical parameters that may indicate the status of the beehive were identified as: temperature, humidity, Carbon Dioxide (CO_2_) levels, and Oxygen (O_2_) levels [32,33]. These are known to vary in response to one or more of these scenarios: the number of honeybees in the hive, the health of the colony, and the external weather conditions [9].

As the proposed approach aims to reduce the power consumption of the power hungry sensors, the data collected with the CO_2_ sensor (as the most power hungry sensor) were selected for focus over the 14 days of the deployment. This means that the EASA was performed on the CO_2_ data as being one of the most important gas sensors in the hive that would give the most significant information about the beehive condition. The platform used for deployment (II) is an off the shelf WSN platform, Libelium “Waspmote” v1.2. It is a low power platform based on Atmega microcontroller, with a modular architecture allowing a combination of over 70 sensors and 11 radio technologies, with a built in SD card slot and an accelerometer. The gas sensors selected for the actual deployment were off the shelf CO_2_ and O_2_ gas sensors that could be interfaced with the gas sensor board developed by Libelium. Additionally, an NO_2_ sensor and two air contaminants sensors were deployed. The actual sensors used in the deployment are: molecular oxygen (O_2_) SK-25; carbon dioxide (CO_2_) TGS4161; nitrogen dioxide (NO_2_) MiCS-2710; and two air contaminants TGS2600 and TGS2602, which sense gases including ethanol (CH_3_CH_2_OH), hydrogen sulfide (H_2_S), and methane (CH_4_). The base station used for this deployment was a bridge between the ZigBee network and the 3G network. It used an XBee Series 2 ZigBee radio and a GSM/GPRS/3G module to send the data to a server. The main issue that was encountered in this deployment was the long sampling period of each of the four sensors and hence very high energy consumption of the system during the sampling state. The results of this deployment have shown that even using an energy harvesting source (*i.e.*, solar) does not guarantee a long lasting WSN node. For this reason, these deployments will benefit significantly from the adaptive sampling using the EASA. The five sensors and the battery in deployment (II) were sampled every 4 h, which translates to a sampling rate of 0.000069 Hz (or 1 sample every 14,400 s).

## 5. Experimental Results

To evaluate the energy consumption of the sensor node in different states, power consumption has been measured. Table 1 and Table 2 present the power consumption and energy consumption of each of the states, together with the relevant task duration, for the two deployments respectively. To characterize the power consumption of the Waspmote platform in different states, a full power characterization was performed using a DC Power analyzer (Agilent N6705B DC) logging data for the current consumption of the platforms while running the firmware described in detail in the text below. Knowing the duration of each state allowed us to calculate the number of samples acquired by the power analyzer and then calculate the average current consumption and energy spent in each of the individual states described below.

In both deployments, the energy consumed during some of the tasks is dependent upon: the sampling rate of the sensor, which changes according to the dynamics of the wind speed (in I), and CO_2_ levels (in II); and also the energy available in the system. An energy model used in the simulations was derived using the measured results presented in Table 1 and Table 2 and Equations (5)–(7) presented in previous sections once the period *T* is known, which, as mentioned earlier, is related to the sampling rate *f_sampling_*.

In deployment (I), the time *t_ACTIVE_* is equal to the sum of the time for sampling the sensors (10 s), communication time between the sensor and the MCU (RS485-UART) and the wireless communication which includes sending and receiving the 16 byte long messages. The data transfer speed of the transceiver CC2520 is 250 kbps and the transmit power is fixed to +5 dBm. The duration of this task is directly related to the number of bytes in the message.

The average power extractable from the airflow can be calculated using Equation (8).

(8) $$P_{Wind}=\frac{1}{2}\eta \;A\;\rho c_{p}\vartheta_{AVG}^{3}$$

where *A* is the swept area of the blades, *ρ* is the density of air, *η* is the efficiency of the rectifier and the DC-DC converter, *v_avg_* is the measured average wind speed and *c_p_* is the power coefficient, which is a function of the tip-speed ratio (*TSR*) of the wind turbine [34]. *c_p_* is determined using both analytical formulas and measurements in the lab with controlled wind-speed and direction. In general it is less than 0.1 for small size turbines. Because the power factor is a function of the *TSR,* it is indirectly a function of the wind speed. In that case the *c_p_* factor cannot have fixed value and must be corrected for different values of the wind speed. The manufacturers of wind turbines usually give a *TSR vs. c_p_* curve. This curve was not available for this work and a characterization of the turbine had to be performed. The characterization was conducted experimentally by measuring the *RPM* and the power produced at different wind speeds; whereas the power available in the wind, and the power factor were calculated using Equations (8) and (9).

**Figure 4:**
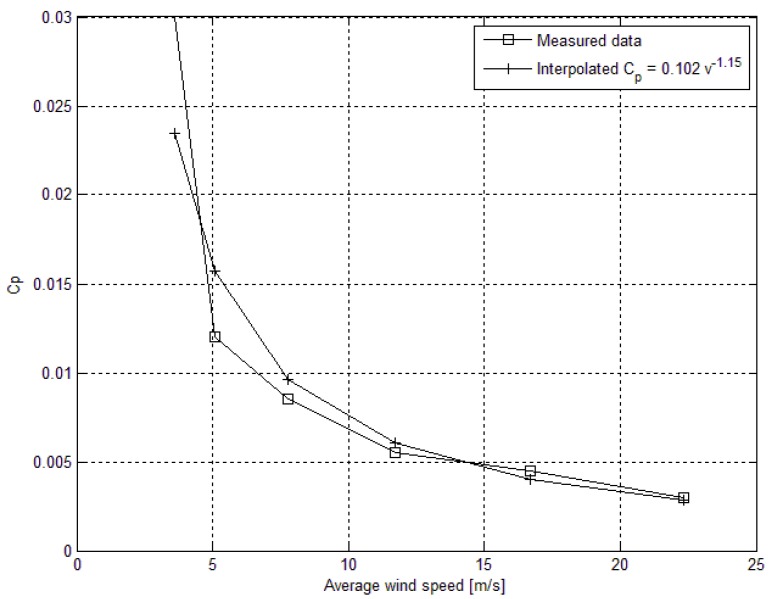
Average wind speed *vs.* power factor *c_p_*.

Figure 4 describes the curve that was generated from the experiment. To be able to use this in the simulation, an interpolation of the measured data was performed. The formula derived with the interpolation was used to correct the *c_p_* factor Equation (10).

(9) $$c_{p}=\frac{P_{out}}{P_{Wind}}$$

(10) $$c_{P}=0.102v_{avg}^{-1.15}$$

When calculating the power extractable from the wind, the wind turbine cut in speed has to be taken into account. For the selected wind turbine the cut in speed was measured to be 3 m/s.

Because the wind speed in deployment (I) was an average wind speed over a period of 10 s, the harvested energy calculated in the simulation for this case must also be for intervals of 10 s. Hence, the energy consumed (*E_consumed_*) in the simulation for every cycle was calculated over a period *T* of 10 s using Equation (11). Finally the total energy available in the system at each time (*t*) is defined by Equation (7) where *E_LEVEL_* is the available energy in the battery after every cycle

*E_consumed_ = E_ACTIVE_* (*T f_EASA_*) + *P_SLEEP_* (*T −* (*T f_EASA_*)) (11)

**Figure 5:**
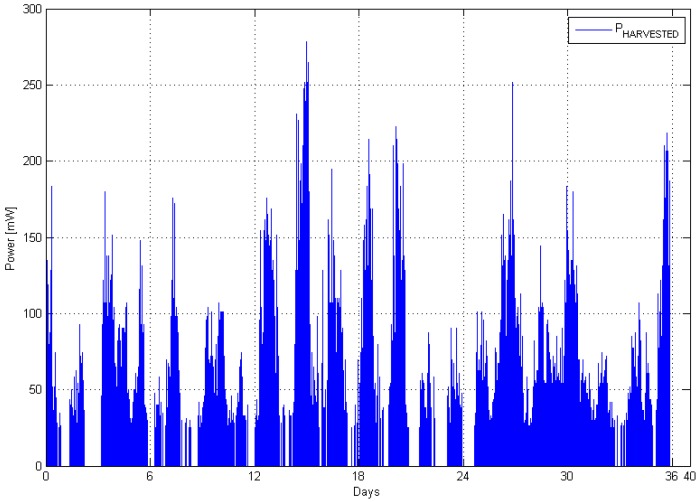
Harvested power from the wind during the 35 days of experiment—deployment (I).

Figure 5 shows the estimated power in the wind calculated using Equation (8) with the measured average wind speed data from deployment (I) and Equation (10) for calculating of the power factor. The values of the rest of the parameters were as follows: efficiency was set to η = 0.56, which reflects the efficiency of the DC-DC conversion and the AC-DC rectifier inside the wind turbine. The density of the air was taken to be *ρ* = 1.2 kg/m^3^ (at *T* = 15 °C).

**Figure 6:**
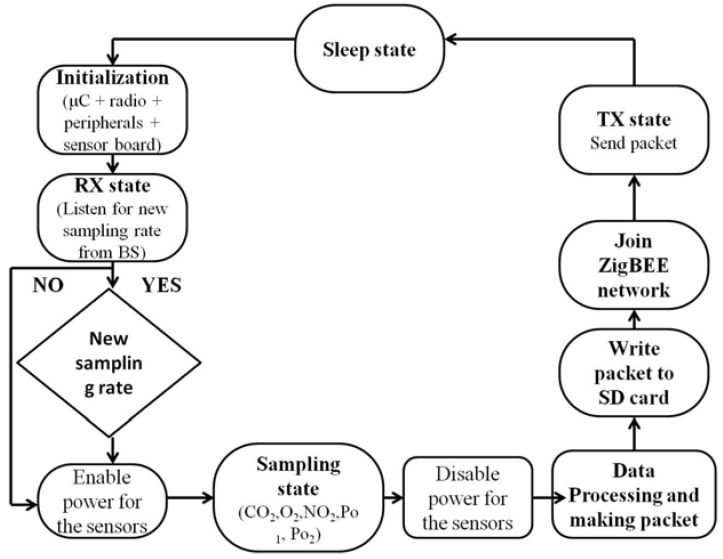
State machine for platform used in deployment (II).

To be able to precisely replicate the energy profile of the Waspmote in deployment (II), an analysis of the energy consumption of the system in various operational states was conducted. A state machine representation of the simplified operation was developed and implemented (Figure 6) replicating the firmware used in deployment (II).

Table 2 summarizes the results of the power consumption analysis of deployment (II). It shows the average current and energy consumption of the Waspmote in different states of its operation, together with the duration of each of the states in deployment (II). The current profile is obtained using an Agilent N6705B DC Power Analyzer configured to sample the current consumption of the node every 5 ms. The figures for the *I_AVG_* in each state are obtained by averaging the measured current over the duration of the individual states.

In deployment (II), the time *t*_ACTIVE_ is equal to the sum of the duration of each of the states. Due to the additional states in deployment (II), Equation (4) has to be rewritten such that includes all of the states described in Figure 6:

(12) $$t_{ACTIVE}=t_{TX}\;+\;t_{RX}+t_{SensorSamp}+t_{PROC}+t_{SD}+\;t_{ZB}+t_{INIT}$$

where $t_{SensorSamp}=\sum_{i=1}^{n}t_{i\;sensor}$ is the sum of the sampling times for all of the sensors, *t_SD_* is the time to write the message to a SD card and *t_ZB_* is the time to join the ZigBEE network.

The total active time (*t_ACTIVE_*) for the microcontroller to perform all of the tasks in this deployment was 173 s. The sleep period (*t_SLEEP_*) was set to be 10 s which makes the total period *T* for the measurement scenario to be *T* = 183 s.

Due to the long sampling period of the sensors, the energy required for sampling the five sensors (Table 2), has the most significant effect on the overall energy consumed by an application duty cycled node. This is purely due to the associated warm up period, which is 41 s for four of the sensors and 1.6 s for the O_2_ sensor.

The energy harvested by the solar panel in deployment (II) was estimated through the information of the battery level, which was acquired with measuring the battery voltage. The period *T* for deployment (II) was 14,400 s. Having the data of the battery level for every 14,400 s (or every 4 h), and knowing how much energy is spent in the active and the sleep state allows the derivation of a rough figure for how much energy is harvested by the solar panel over the 4 h. Using Equation (11), we can calculate the consumed energy after each cycle *T*. Using Equation (12), the time taken to execute all of the sub states of the active state which for deployment (II) was 173 s. Subtracting this time from the period *T* will give the period spent in sleep state (*t_SLEEP_* = 14,227 s). If it is assumed that the battery has capacity *C* (mAh), the battery level at moment t seconds was *X*(*t*) (in %), and after 4 h the level is *X*(*t* + 14400), then the harvested energy with the solar panel could be estimated using Equation (13):

(13) $$E_{harvested}=(X\left(t+14400\right)-X\left(t\right))\frac{C}{100}-E_{consumed}$$

If the result from Equation (13) is negative, we assume that no energy has been harvested. The power harvested is then calculated using Equation (14).

(14) $$P_{harvested}=\;\frac{E_{harvested}}{T}$$

**Figure 7:**
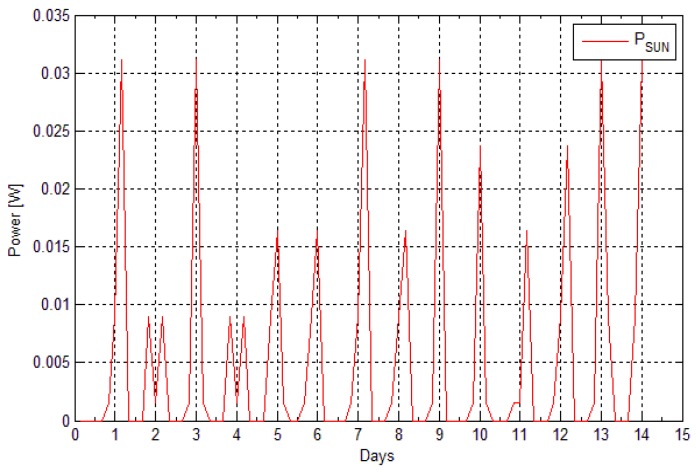
Harvested power from sun—deployment (II).

Figure 7 gives the estimated power harvested by the solar panel for the days of deployment (II), derived using Equations (13) and (14). The battery used for both the calculations and the deployment (II) had a capacity of 800 mAh. The solar panel had the following specifications: *P_MAX_* = 1.3 W, *V_MAX_* = 6.5 V, *I_MAX_* = 205 mA and dimensions of 0.111 m × 0.091 m.

## 6. Simulation Results and Discussion

The state machines presented in Figure 1 and Figure 6 were implemented and executed in MATLAB, following the node energy model described by the equations in Section 5, and using the infield power consumption measurements and sensor data from the both deployments.

For deployment (I), Equation (5) was used to calculate the energy spent in the active state. Using Equation (11) the energy spent after each cycle *T* was calculated, and using Equation (7) the energy available in the battery after each cycle was found. The cycle was *T* = 10 s. In this case we analyze a custom made WSN node with only one energy harvesting source (*i.e.*, wind) and one power hungry sensor (*i.e.*, ultrasonic wind sensor). The harvested energy was calculated using a real field data and Equations (3) and (8).

For the deployment (II), the energy model is described with Equations (7), (11) and (12). In this case, we analyze an off the shelf WSN node with five power hungry gas sensors that require long warm up periods. From an energy harvesting prospective, in this case, we have one energy source (*i.e.*, solar). The harvested energy was estimated through the battery level using Equation (13) with the methodology described in Section 5. In both the simulations we assume that the ASA is running on the base station evaluating, for each period, the maximum frequency. The node is capable of recharging its battery by harvesting energy from the wind or sun, respectively, with the scaled wind turbine described in Section 4 and the solar panel described in Section 5. We also assume that the nodes are aware of the available energy. The EASA was implemented in MATLAB with the following values for its relevant parameters: *c* = 2.1, *h* = 5, *W* = 400, *δ* = 0.1% and *X_level_* = 70%. *X_level_* is the critical level of the battery at which the sampling rate is adapted according to Equations (1) and (2). Finally, the capacity of the battery in both of the deployments is 800 mAh.

**Figure 8:**
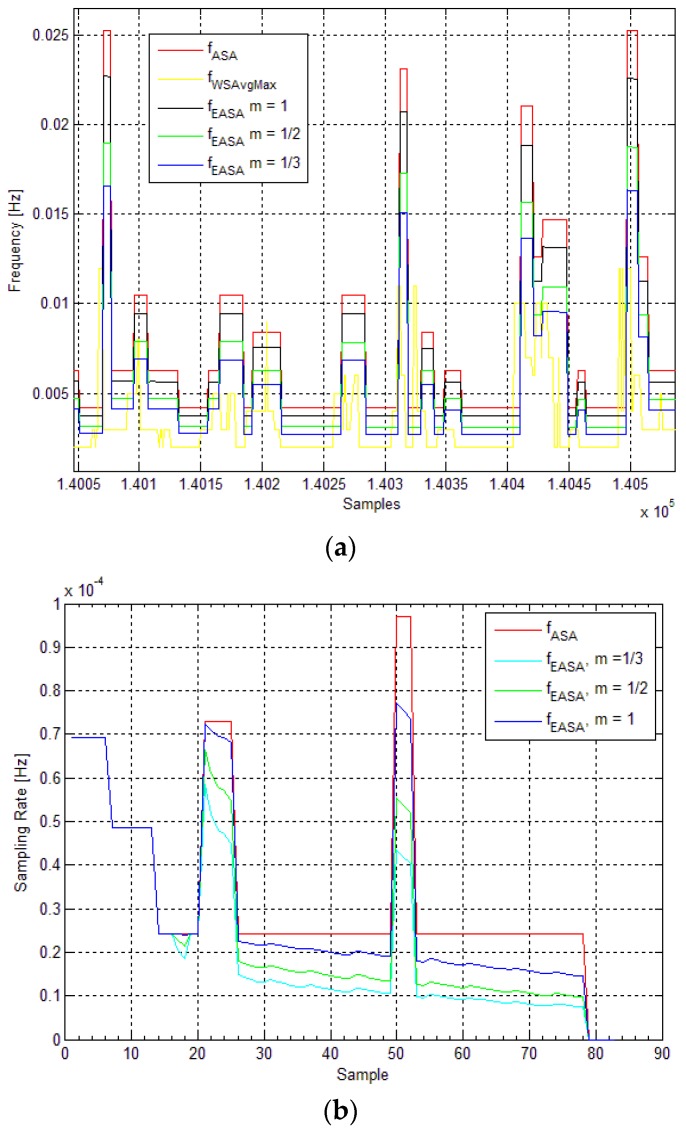
(**a**) Deployment (I): Sampling rates and maximum frequency of the average wind speed signal *vs.* samples; (**b**) Deployment (II): Sampling rates and maximum frequency of the average wind speed signal *vs.* samples.

Figure 8 shows the sampling rate of ASA (*f_ASA_*) *vs.* the sampling rate of the EASA (*f_EASA_*) for deployment (I) and (II), respectively, for different values of *m*. In Figure 8a, the maximum frequency of the measured average wind speed is also presented. In both cases (Figure 8a,b), the sampling rate of ASA is adjusted by the energy aware routine, according to Equations (1) and (2). As mentioned earlier, a potential disadvantage of the EASA is the possibility aliasing occurring. When the energy level of the node is critical, the EASA could reduce the sampling frequency (rate) to a level at which it will no longer comply with the *Nyquist* criterion. This could cause under-sampling of the sensor signal to occur, in which case the maximum frequency of the signal will be greater than half the sampling rate. Such event is more likely to happen when the EASA is more conservative, which depends on the value of the parameter *m*. As depicted in Figure 8a, in the case when *m* = 1/3, in some occasions, the maximum frequency of the measured signal (wind speed) is higher than the sampling rate of EASA. This will introduce an error in the measurement, which could be taken as a trade-off for staying active in the network, rather than continuing to sample at full rate and discharging the battery completely.

**Figure 9:**
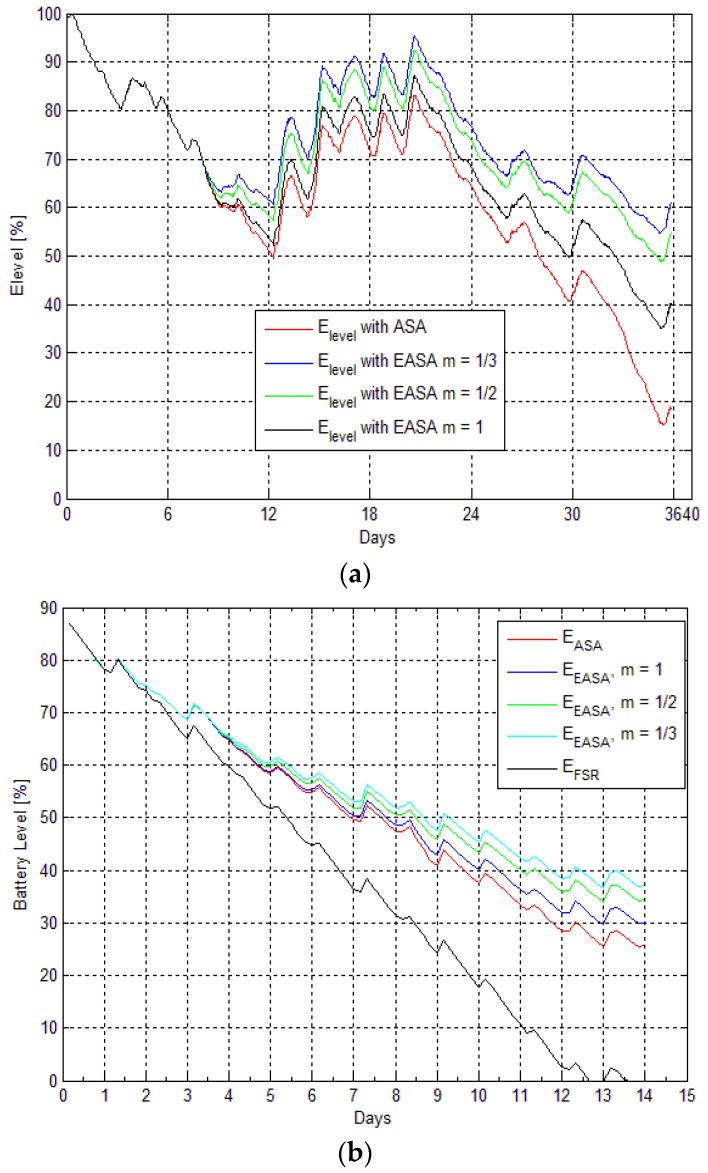
(**a**) Deployment (I): Energy in the system (ASA *vs.* EASA) as a function of time; (**b**) Deployment (II): Energy in the system (ASA *vs.* EASA) and fixed sampling rate energy level (*E_FSR_*) as a function of time.

In Figure 9, a comparison of the energy profiles of the nodes is presented. For both deployments (I) and (II), we consider the following cases: (i) the node is using the sampling rate from ASA; and (ii) when the node is adjusting the sampling frequency according to its available energy using Equations (1) and (2) with three different values of the parameter *m*. For deployment (II), the energy profile of the node with fixed sampling rate *E_FSR_* is also presented. In both cases (Figure 9a,b), when the energy level is above the threshold level (*X_level_ =* 70%), the sampling frequencies (rates) remain the same (*f_EASA_ = f_ASA_*) and the energy levels are identical, which means that the energy consumed in both cases is same. When the energy level is below *X_level_* the sampling frequencies are no longer the same, which is reflected in the energy consumed by the nodes. The node on which EASA is running saves energy by reducing its sampling rate according to the current energy level. If the sampling frequency is not adapted to the energy available in the battery, the energy levels can significantly drop (below 20% in (Figure 9a) and to 0% in (Figure 9b)). In both of the figures, the same trend is present where the node running the ASA and the node using the EASA have identical energy levels until the critical level *X_level_* is reached. In this figure the difference between the energy levels of node sampling the sensors with fixed sampling rate (FSR) and harvesting energy from only one source (*i.e.*, sun in this case) can be noticed. This confirms that a significant savings can be achieved using the EASA, in comparison with using a fixed sampling rate strategy.

## 7. Conclusions

In this paper, a novel Energy Aware Adaptive Sampling Algorithm for WSN with power hungry sensors and energy harvesting capability is presented. It has been shown that combining a traditional approach for adaptive sampling and energy aware management technique performed at node level can save energy, facilitating self-sustainability through energy harvesting. When the battery level is above a critical level, defined by the user (*X_level_*), the proposed algorithm can use the sampling rate of any ASA. As the battery level goes below the critical level, the algorithm becomes more energy conservative by reducing the sampling rate. Thus, the node manages its activity in the network according to its energy levels. The algorithm was validated using real in-field data from two deployment scenarios in two different application spaces, namely, measuring wind speed and direction and monitoring bees in a smart beehive. These scenarios involved different sensors, including ultrasonic anemometers and gas sensors. Moreover, two different energy harvesting sources are considered, namely wind and solar. The aim is to achieve a self-sustainable system that can manage its activity according to the available energy. The error introduced due to aliasing can be accepted as a trade-off for staying active in the network, and not discharging the battery completely. Using the combination of energy harvesting, adaptive sampling algorithm, and energy aware management technique, an end user can be guaranteed a self-sustainable wireless sensor network solution. The presented approach is especially suitable for applications with energy hungry sensors, and applications where maintenance has to be reduced to a minimum.

## Figures and Tables

**Table 1:** Power and energy consumption for various tasks (figures taken from datasheet).

Task	Power (mW)	Time (ms)	Energy (µJ)
Data Transmission (16 bytes)	108.9 ^a^	*t*_TX_ = 0.5	54.45
Data Receiving (16 bytes)	59.4 ^a^	*t*_RX_ = 0.5	29.7
Wind Sensor	36 ^b^	10,000	360,000
MCU active	0.858 ^c^	*t*_ACTIVE_ = 10,000 + *t*_PROC_ + *t*_TX_ + *t*_RX_	8584.29
MCU sleep	0.0129 ^c^	*T* – *t*_ACTIVE_	N/A
Wind Sampling (MCU active + Wind Sensor)	36.858	10,000	368,580
MCU sleep +Radio sleep + Wind Sensor off + other ICs	0.055 ^d^	*T* – *t*_ACTIVE_	N/A

^a^ CC2520 datasheet figures; ^b^ Average power consumption of WMT52; ^c^ MSP430F5437A datasheet figures; ^d^ Measured sleep current of the platform @ 3.7 V Isleep = 15 μA.

**Table 2:** Measured average current consumption of the Waspmote in different states at supply voltage of 3.7 V.

State	*I*_AVG_ (mA)	Time (s)	Energy (mJ)
Sleep	2	*T*-*t*_ACTIVE_ = 10	74
Initializations of sensor board and configuration of uC	112.7	*t*_INIT_ = 5	2084.95
Sampling CO_2_	190.6	*t*_CO2_ = *41*	28,914.02
Sampling NO_2_	156	*t*_NO2_ = 41	23,665.2
Sampling O_2_	115.1	*t*_O2_ = 1.6	681.392
Sampling Pollution sensor 1	113.2	*t*_PO1_ = 41	17,172.44
Sampling Pollution sensor 2	83.1	*t*_PO2_ = 41	12,606.27
Send packet (82 bytes)	40	*t*_TX_ = 0.0026	0.3848
Receive packet (82 bytes)	40	*t*_RX_^a^ = 0.0026	0.3848
Write packet to SD card	45	*t*_SD_ = 0.017	2.8305
Processing (SPI comm., ADC *etc.*)	30	*t*_PROC_ = 0.002	0.222
Join ZigBee network	50	*t*_ZB_ = 3	555

^a^ This time can be reduced and depends on the length of the message from the BS containing the sampling rate from ASA. In our case the time in TX and RX are same.

## Abbreviations

The following abbreviations are used in this manuscript:

WSN	Wireless Sensor Networks
ASA	Adaptive Sampling Algorithm
EASA	Energy Aware Adaptive Sampling Algorithm
MAC	Medium Access Control
SHM	Structural Health Monitoring
MCU	Micro Controller Unit
BS	Base Station
ADC	Analog to Digital Converter
RISC	Reduced Instruction Set Computer
CPU	Central Processing Unit
SRAM	Static Random Access Memory
DC-DC	Direct current to Direct current
SD	Secure Digital
GSM	Global System for Mobile Communication
GPRS	General Packet Radio Service
3G	Third Generation mobile telecommunication technology
UART	Universal Asynchronous Receiver/Transmitter
TSR	Tip Speed Ratio
FSR	Fixed Sampling Rate

## Appendix

The used notation are shown in Table A1.

**Table A1:** Used notation.

*f_Max_*	Maximum frequency of a sensor signal
*f_Sampling_*	Sampling rate of a sensor
*W*	Initial number of sensor samples
*c*	Confidence parameter
*f_Down_*	Lower frequency borders
*f_Up_*	Upper frequency borders
*δ*	Minimum percentage change in the maximum frequency of a sensor signal
*h*	Consecutive number of samples after which the ASA detects a change
*f_Curr_*	Current sampling rate of a sensor
*f_EASA_*	Sampling rate of EASA
*E_batt_*	Energy level in a battery
*X_level_*	Critical level of a battery in percentage
*K*	Cost function parameter
*m*	Cost function parameter
*E_ALG_*	Energy spent by MCU to execute an algorithm
*P_MCU_*	Power consumption of a MCU
*t_ALG_*	Time required to execute an algorithm
*t_iharv_*	Period of harvesting energy of source *i*
*P_i HARVESTED_*	Harvested power by source *i*
*t_ACTIVE_*	Active time of a node
*t_RX_*	Time spent in receive mode
*P_RX_*	Power spent in receive mode
*P_SensorSampling_*	Power spent sampling a sensor
*P_PROC_*	Power spent processing data
*t_PROC_*	Time spent processing data
*t_TX_*	Time spent in transmit mode
*P_TX_*	Power spent in transmit mode
*t_SLEEP_*	Time spent in sleep mode
*P_SLEEP_*	Power spent in sleep mode
*E_LEVEL_*	Current energy level after each cycle
*E_SLEEP_*	Energy spent in sleep mode
*I_sleep_*	Sleep current of a platform
*P_WIND_*	Average Electrical power extractable from the airflow
*ρ*	Air density
*η*	Efficiency of a rectifier and a DC-DC converter
*v_avg_*	Measured average wind speed
*c_p_*	Power coefficient of a turbine
*A*	Swept area of the blades of a turbine
*P_OUT_*	Measure electrical power output of a turbine
*E_consumed_*	Energy consumed after one cycle
*T*	Period of one cycle
*t_ZB_*	Time to join a ZigBEE network
*t_SD_*	Time to write a message to a SD card
*t_SensorSamp_*	Time to sample a sensor
*t_iSensor_*	Time to sample sensor *i*
*t_INIT_*	Time to initialize a platform
**t_*CO*2_	Time to sample a CO_2_ sensor
*t*_*NO*2_	Time to sample a NO_2_ sensor
*t*_*O*2_	Time to sample an O_2_ sensor
*I_AVG_*	Measured average current consumption of a WASP mote in different states
*t*_*PO*1_	Time to sample a pollution sensor 1
*t*_*PO*2_	Time to sample a pollution sensor 2
*C*	Capacity of a battery
*P_MAX_*	Maximum rated power of a solar panel
*V_MAX_*	Maximum rated voltage of a solar panel
*I_MAX_*	Maximum output current of a solar panel
*E_FSR_*	Energy spent by a platform using fixed sampling rate
